# Genome-Wide Expression of Azoospermia Testes Demonstrates a Specific Profile and Implicates *ART3* in Genetic Susceptibility

**DOI:** 10.1371/journal.pgen.0040026

**Published:** 2008-02-08

**Authors:** Hiroyuki Okada, Atsushi Tajima, Kazuyoshi Shichiri, Atsushi Tanaka, Kenichi Tanaka, Ituro Inoue

**Affiliations:** 1 Department of Obstetrics and Gynecology, Niigata University Graduate School of Medical and Dental Sciences, Niigata, Japan; 2 Division of Molecular Life Science, School of Medicine, Tokai University, Isehara, Japan; 3 Department of Obstetrics and Gynecology, Tachikawa Hospital, Nagaoka, Japan; 4 St. Mother's Hospital, Kitakyushu, Japan; 5 Core Research for Evolutional Science and Technology, Japan Science and Technology Corporation, Kawaguchi, Japan; The Wellcome Trust Sanger Institute, United Kingdom

## Abstract

Infertility affects about one in six couples attempting pregnancy, with the man responsible in approximately half of the cases. Because the pathophysiology underlying azoospermia is not elucidated, most male infertility is diagnosed as idiopathic. Genome-wide gene expression analyses with microarray on testis specimens from 47 non-obstructive azoospermia (NOA) and 11 obstructive azoospermia (OA) patients were performed, and 2,611 transcripts that preferentially included genes relevant to gametogenesis and reproduction according to Gene Ontology classification were found to be differentially expressed. Using a set of 945 of the 2,611 transcripts without missing data, NOA was further categorized into three classes using the non-negative matrix factorization method. Two of the three subclasses were different from the OA group in Johnsen's score, FSH level, and/or LH level, while there were no significant differences between the other subclass and the OA group. In addition, the 52 genes showing high statistical difference between NOA subclasses (*p* < 0.01 with Tukey's *post hoc* test) were subjected to allelic association analyses to identify genetic susceptibilities. After two rounds of screening, SNPs of the ADP-ribosyltransferase 3 gene (*ART3*) were associated with NOA with highest significance with ART3-SNP25 (rs6836703; *p* = 0.0025) in 442 NOA patients and 475 fertile men. Haplotypes with five SNPs were constructed, and the most common haplotype was found to be under-represented in patients (NOA 26.6% versus control 35.3%, *p* = 0.000073). Individuals having the most common haplotype showed an elevated level of testosterone, suggesting a protective effect of the haplotype on spermatogenesis. Thus, genome-wide gene expression analyses were used to identify genes involved in the pathogenesis of NOA, and *ART3* was subsequently identified as a susceptibility gene for NOA. These findings clarify the molecular pathophysiology of NOA and suggest a novel therapeutic target in the treatment of NOA.

## Author Summary

Worldwide, approximately 15% of couples attempting pregnancy meet with failure. Male factors are thought to be responsible in 20%–50% of all infertility cases. Azoospermia, the absence of sperm in the ejaculate due to defects in its production or delivery is common in male infertility. In this study, we focused on non-obstructive azoospermia (NOA) because the etiologies of obstructive azoospermia are well studied and distinct from those of NOA. Microdeletions of the Y chromosome are thus far the only genetic defects known to affect human spermatogenesis, but most cases of NOA are unsolved. Because NOA results from a variety of defects in the developmental stages of spermatogenesis, the stage-specific expressions of genes in the testes must be investigated. Thus, genome-wide gene expression analyses of testes of NOA can provide insight into the several etiologies and genetic susceptibilities of NOA. In the present study, we analyzed several differentially expressed genes in NOA subclasses and identified ART3 as a susceptibility gene for NOA.

## Introduction

Spermatogenesis, a major function of mammalian testes, is complex and strictly regulated. While spermatogenesis is a maturation of germ cells, other cells including Sertoli, Leydig, and peritubular myoid cells also play important roles, and defects at any differentiation stage might result in infertility. Male infertility is estimated to affect about 5% of adult human males, but 75% of the cases are diagnosed as idiopathic because the molecular mechanisms underlying the defects have not been elucidated. In consequence, an estimated one in six couples experiences difficulty in conceiving a child despite advances in assisted reproductive technologies. Male-factor infertility constitutes about half of the cases, and a significant proportion of male infertility is accompanied by idiopathic azoospermia or severe oligozoospermia, which may well have potential genetic components. It is well-recognized that men with very low sperm counts (<1 million/ml), identified through an infertility clinic, have a higher incidence of Y-chromosome microdeletion (up to 17%) [1,2]. However, the genetic causalities of most cases of azoospermia are not known.

Global gene-expression profiling with microarray technologies has been applied with great promise to monitor biological phenomena and answer biological questions. Indeed, microarray technologies have been successfully used to identify biomarkers, disease subtypes, and mechanisms of toxicity. We applied microarray analysis to testis specimens from infertile individuals including patients with obstructive azoospermia (OA) and non-obstructive azoospermia (NOA [OMIM %606766]) to characterize NOA and to identify the specific pathophysiology and molecular pathways of the disease. In addition, we attempted to identify genetic susceptibility to NOA from genes differentially expressed in NOA testes.

Genes related to spermatogenesis and candidate genes for azoospermia have been surveyed in humans and mice, especially since gene targeting technology accelerated the identification of genes that play crucial roles in spermatogenesis [3]. Because spermatogenesis is a complex process including meiosis, a germ cell–specific event, gene expression profiles specific to the differentiation stage, clinically classified by the Johnsen's score, were examined to provide insight into the pathogenesis of azoospermia [4]. In the current study, we performed microarray analyses on biopsied testes obtained from 47 NOA patients at diverse clinical stages without prior selection and 11 OA patients. The 47 NOA samples showed a wide range of heterogeneity, including a series of impairments at the differentiation stage of spermatogenesis that so far have been evaluated mainly by pathological findings. Thus, classification of NOA at the transcriptome level is a necessary first step in elucidation of the molecular pathogenesis of NOA. To do this, we adopted the non-negative matrix factorization (NMF) method, an unsupervised classification algorithm developed for decomposing images that has been applied in various fields of science including bioinformatics because of its potential for providing insight into complex relationships in large data sets [5–7]. 47 of the NOA-samples were divided into three subclasses by the NMF method, and each class was associated with clinical features. 149 transcripts were identified as differentially expressed genes among the NOA subclasses according to a statistical criterion, and the features involved in spermatogenesis based on Gene Ontology classification were demonstrated.

The genetic causality of NOA most likely involves the expression level of a susceptibility gene, which might be detected by genome-wide gene expression analysis. While it is daunting to identify genetic susceptibility from 100-1000 differentially expressed genes, genetic susceptibility might more readily be identified from random genes differentially expressed with high significance rather than by investigating only genes in a specific biological pathway. Based on a well-defined statistical procedure, 52 candidate genes for NOA were catalogued by gene expression profile and screened for allelic association study in a total of 442 NOA patients and 475 fertile male controls. After gene-centric selection of SNPs, 191 SNPs of 42 candidate genes were initially evaluated for allelic association with NOA. After two rounds of screening, SNPs of the ADP-ribosyltransferase 3 (*ART3*) gene were found to be significantly associated with NOA, and five of these SNPs were selected for haplotype construction. The most common haplotype was significantly under-represented in the patients and may be protective. The functional impact of this haplotype was further investigated.

## Results

### Extraction of NOA-Related Gene Expression Profile

**Figure 1:**
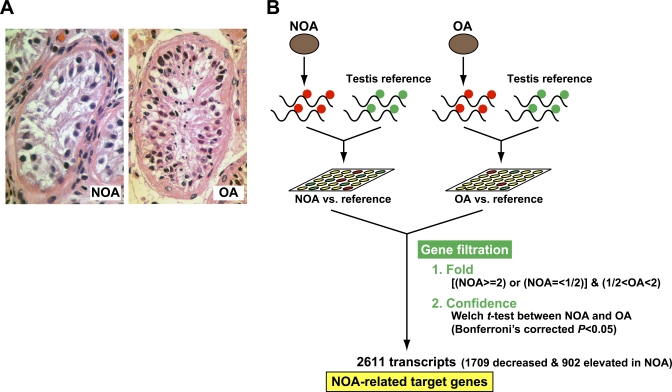
Experimental Design to Extract NOA-related Target Genes (A) Representative testicular biopsies from NOA (left panel) and OA (right panel) patients. The seminiferous tubules in NOA testis show Sertoli cells only; testicular histology of the OA patient indicates the presence of germ cells at all stages of spermatogenesis. (B) Strategy for discovering the NOA-specific expression profile, i.e., the NOA-related target genes. Compared with the expression level of reference RNA, the NOA group, with expression undergoing 2-fold or more mean change, was extracted; the OA group comprised transcripts with less than 2-fold mean expression change. Of the overlapping transcripts, only those with statistically significant difference between NOA and OA (*p* < 0.05 with Bonferroni's correction) were identified as differentially expressed.

As shown in Figure 1A, the most notable difference in histological findings between NOA and OA testes was that the NOA patients exhibited, at varying degrees, incomplete sets of spermatogenic germ cells (spermatogonia, spermatocytes, spermatids, and spermatozoa) in the seminiferous tubules. In severe NOA patients, we could not even detect Sertoli cells, the major somatic cells of the seminiferous tubules, on histological examination (figure not shown), indicating clinical heterogeneity of NOA testes. In order to elucidate the molecular systems underlying NOA at the transcriptome level, it is important to extract genes reflecting the diversity of NOA phenotypes. For this purpose, we first compared global gene expression profiles in NOA testes to those of OA testes using the Agilent Human 1A(v2) Oligo Microarray system. We chose the ‘standard reference design' in two-color microarray experiments as an experimental design for the expression analysis [8], where a single microarray was used to compare either NOA or OA to the testicular reference RNA as described in Materials and Methods (Figure 1B).

**Figure 2:**
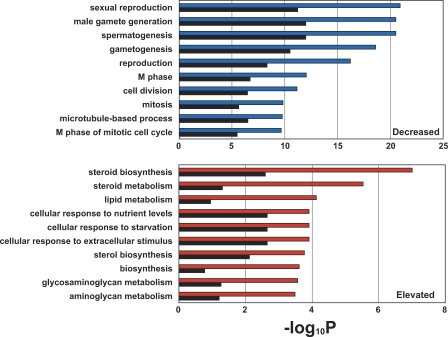
Comparison of Ten Top-ranked Gene Ontology (GO) Categories Significantly Over-Represented in the Two Subsets (1,709 Decreased, Upper Panel; 903 Elevated, Lower Panel) of NOA-related Target Genes Blue and red bars represent *p*-values for the ten top-ranked GO categories over-represented in the decreased and elevated gene lists, respectively. Black bars show the corresponding *p*-value using 2,611 NOA-related target genes. *p*-Values were determined by Fisher's exact test using all of the GO-annotated genes on the Agilent Human 1A(v2) microarray as a background, as shown in Table 1. The *p*-values are expressed as the negative logarithm (base 10).

Of the 18,716 transcripts screened with the microarray, we obtained transcripts that showed a 2-fold mean expression difference between NOA and the reference, the NOA group; the OA group comprised transcripts showing less than 2-fold mean expression difference between OA and the reference (Figure 1B). Of the transcripts overlapping the two groups, 2,611 transcripts were found to be differentially expressed between NOA and OA testes after statistical filtering (based on lowess-normalized natural log[Cy5/Cy3], Bonferroni's corrected *p* < 0.05). This gene list, termed NOA-related target genes, comprised 902 elevated and 1,709 decreased transcripts in NOA testes. To characterize the gene list from the biological aspect, the 2,611 transcripts were subjected to functional clustering according to Gene Ontology (GO) classification for biological processes with GeneSpring software. We identified a total of 190 GO categories that were significantly (*p* < 0.05 without multiple testing correction) over-represented among the 2,611 transcripts. Table 1 shows the ten top-ranked GO categories in descending order of significance based on *p*-values with Fisher's exact test. It is notable that the GO categories involved in gametogenesis (GO:48232; 7283; 7276), reproduction (GO:19953; 3), and the cell cycle (GO:279; 51301; 7049; 7067) are significantly associated with the gene list. We further analyzed two separate subsets comprising 1,709 decreased (Figure 2, upper) and 902 elevated (Figure 2, lower) transcripts, based on their GO annotations. The top-ranked GO categories for NOA-related target genes are more similar to those of the 1,709 decreased transcripts than to those of the 902 elevated ones (Figure 2; Table 1). Thus, the predominant features may reflect spermatogenic defects common to NOA testes. On the other hand, 902 transcripts elevated in NOA testes exhibited a distinct GO profile that included several GO categories involved in biosynthesis and metabolism in cytoplasm (Figure 2), implying an increase in cytoplasmic turnover rates such as steroid turnover in NOA testes.

**Table 1:**
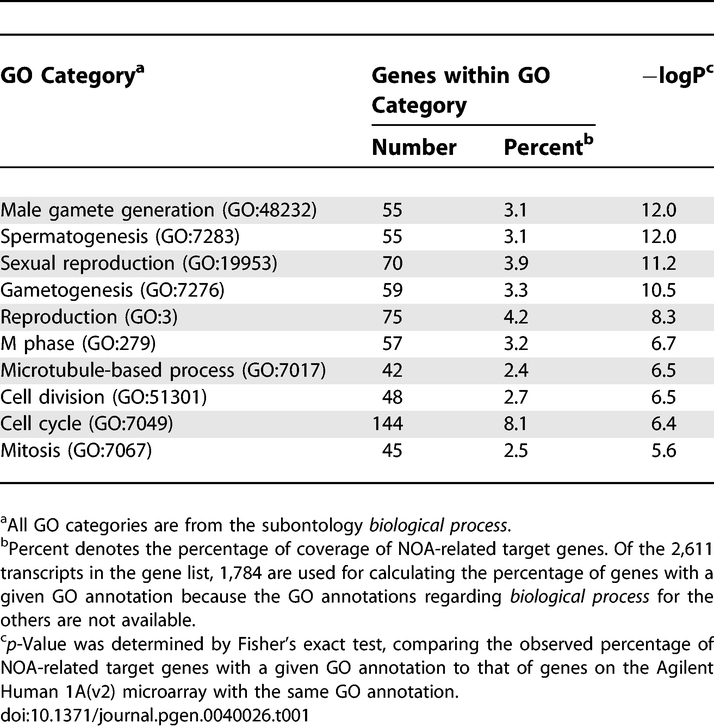
Top-Ranked Ten Categories of Gene Ontology Significantly Overrepresented among 2,611 Transcripts

### Discovery of Three Molecular Subclasses of NOA Testes

**Figure 3:**
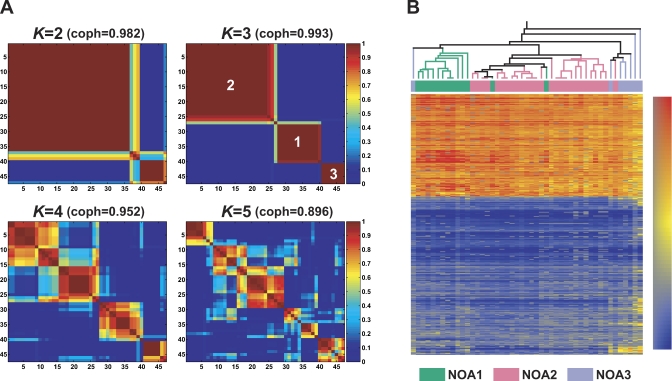
Non-Negative Matrix Factorization (NMF) and Hierarchical Clustering (HC)-based Subclassification of 47 NOA Samples (A) Reordered consensus matrices averaging 50 connectivity matrices computed at *K* = 2–5 (as the number of subclasses modeled) for the NOA data set comprising of NOA-related target genes. NMF computation and model selection were performed according to Brunet et al. [6] as described in Materials and Methods. According to cophenetic correlation coefficients (coph) for NMF-clustered matrices, the NMF class assignment for *K* = 3 was the most robust. (B) The HC method incorporated in GeneSpring also was used to classify NOA heterogeneity in testicular gene expression. To display correspondences of subclassification by the two methods, the NMF class assignments for *K* = 3 are shown color-coded; NOA1 (green), NOA2 (pink), and NOA3 (light purple).

To clarify heterogeneity of NOA testes at the transcriptome level, we further examined NOA-related target genes to identify NOA subclasses without prior classification with pathological features. We adopted the NMF algorithm coupled with a model selection method [6] to a complete dataset of 945 out of the 2,611 transcripts without missing values of signal intensities for a total of 47 NOA samples. Figure 3A shows reordered consensus matrices averaging 50 connective matrices generated for subclasses *K* = 2, 3, 4, and 5. Distinct patterns of block partitioning were observed at models with 2 and 3 subclasses (*K* = 2 and 3), whereas higher ranks (*K* = 4 and 5) made block partitioning indistinct. Thus, the NMF method predicts the existence of reproducible and robust subclasses of NOA samples for *K* = 2 and 3. This prediction was quantitatively supported by higher values of cophenetic correlation coefficients (coph) for NMF-clustered matrices. The NMF class assignment for *K* = 3 showed the highest coph value (coph = 0.993), indicating that three molecular subclasses, termed NOA1, NOA2, and NOA3, are the most reasonable subclassification among 47 NOA samples. For comparative analysis of class discovery, a hierarchical clustering (HC) approach was applied to log-transformed normalized ratios for NOA-related target genes. As shown in Figure 3B, the HC dendrogram exhibited a clustering pattern similar to that of the NMF-based subclassification, as the three NMF-subclasses of NOA samples tended to form distinct clusters in the HC analysis. Thus, the HC clustering for NOA-related target genes appears to justify the three NMF-based subclasses of NOA samples.

To investigate the clinical features of the three NOA subclasses, we compared several clinical measures among the subclasses. The results obtained from statistical analyses in a total of four groups including the OA group are summarized in Table 2. We found significant differences in the three NOA-related clinical characteristics, testicular histological score (Johnsen's score, *p* = 1.4 × 10^−6^), serum FSH level (*p* = 9.8 × 10^−4^), and LH level (*p* = 0.0051) among the four groups using Kruskal-Wallis test, but there were no differences in age and serum testosterone level. Post hoc pairwise comparisons revealed that both the NOA1 and NOA2 groups exhibited low Johnsen's scores and high levels of serum FSH compared with the OA group (Table 2). In the NOA1 group, a high LH level (*p* < 0.01) also was found compared with the OA group. On the other hand, there were no significant differences in any of the parameters between the NOA3 and OA groups, as well as among three NOA subclasses in *post hoc* analysis. Elevations of serum FSH and LH concentrations often are observed in infertile patients with abnormal testicular histologies and are correlated, to some extent, with the severity of spermatogenic defects [9,10]. Testicular histologies of NOA and OA patients have been evaluated by the Johnsen's scores, ranging from 10 to 1 according to the presence or absence of spermatogenesis-related cell types (spermatozoa, spermatids, spermatocytes, spermatogonia, and Sertoli cells) in seminiferous tubules [11]. The NMF-based subclasses of testicular gene expression showed that the low score classes had heterogeneity (NOA1 and NOA2), presumably indicating the possibility of distinct spermatogenic defects at the molecular level that could not be detected by morphological examination.

**Table 2:**
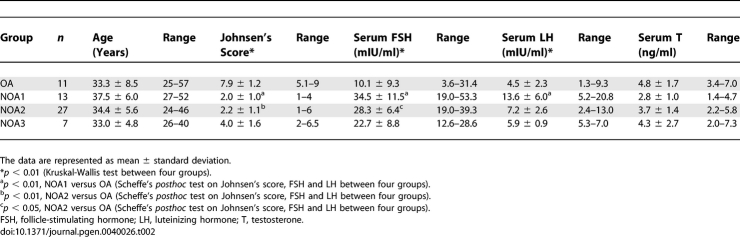
Clinical Characteristics of Three Molecular Subclasses of NOA

### Identification of Transcripts Differentially Expressed in the Three NOA Subclasses

**Figure 4:**
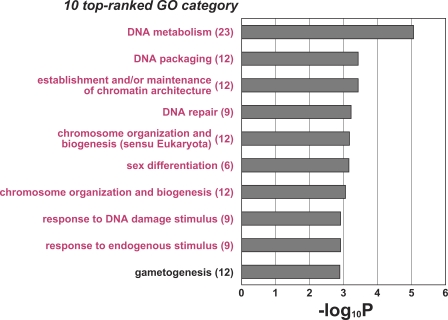
Significant Enrichments of Gene Ontology Categories in GO-based Profiling of 149 Differentially Expressed Transcripts Grey bars represent *p*-values (expressed as the negative logarithm [base 10]) for the 10 top-ranked GO categories over-represented in the 149 differentially expressed transcripts, using the 2,611 NOA-related target transcripts as a background set of genes for the determination of *p*-values. The actual number of differentially expressed transcripts involved in each category is given in parentheses. In the process of extracting the 149 transcripts from the NOA-related target gene list, sets of genes involved in nine GO categories, marked in magenta, were more condensed because they were not found in the previous 10 top-ranked GO lists of NOA-related target genes (see Table 1 and Figure 2).

Based on the three NOA subclasses, we conducted further statistical analyses to extract transcripts representing expression differences between NOA subclasses from the NOA-related target genes (Figure S1). 149 out of 2,611 transcripts showed significant differences (*p* < 0.05, Tukey's *post hoc* test) in testicular expression between the NOA subclasses, as summarized in Table S1. To characterize this gene list based on GO classification for biological processes, we examined which GO terms were highly associated with the 149 differentially expressed transcripts, relative to those for the NOA-related gene list (as shown in Table 1 and Figure 2). Figure 4 shows the 10 top-ranked GO categories for the 149 transcripts, using the 2,611 NOA-related target transcripts as a background set of genes for this GO analysis. Nine GO categories excluding gametogenesis appeared to be novel, indicating that highly significant enrichments of transcripts involved in DNA metabolism (GO:6259; 6325; 6323; 6281), chromosome organization and biogenesis (GO:51276; 7001), sex differentiation (GO: 7548), and response to endogenous stimulus (GO:9719; 6974) occurred after the extraction of 149 transcripts from the NOA-related target gene list (Figure 4). Other features of the 149 transcripts from the gene list (Table S1) were as follows: (1) a high frequency (24.2%) of sex chromosome-linked genes; (2) a high frequency (13.4%) of genes encoding cancer/testis antigens [12,13]; and (3) a moderate frequency (6.7%) of male infertility-related genes. Defect of these genes results in male infertility/subfertility in mice [3,14–16].

Twenty-five of the 149 transcripts showing differences in between-subclass expression displayed elevated expression in NOA, while the others (124 transcripts) had decreased expression (Table S1). The 25 NOA-elevated transcripts accounted only for differences in testicular expression between NOA1 and the other two subclasses, NOA2 and NOA3 (Figure S2; Table S1), suggesting testicular hyperactivity in NOA1 patients. For example, 3β-hydroxysteroid dehydrogenase, encoded by *HSD3B2* and *HSD3B1*, plays a crucial role in biosynthesis of testosterone in Leydig cells [17]. Expression levels of the two transcripts in the NOA1 subclass were higher than those in the NOA2 and NOA3 subclasses, and the expression difference between NOA1 and NOA3 was significant by Tukey's *post hoc* test (Figure S2; Table S1). As the NOA1 subclass showed significantly high LH and slightly low testosterone levels (Table 2), the elevated levels of the two transcripts may be explained by a compensation process for maintaining normal testosterone level. Thus, such enhanced steroidogenesis of the NOA1 subclass might favor, even if only slightly, testicular hyperactivity in NOA1 patients.

On the other hand, among the 124 NOA-decreased transcripts, most transcripts (118/124) showed expression differences between NOA3 and the other two subclasses (Figures S2–S4; Table S1). Expression levels of these transcripts in the NOA3 subclass were similar to those in testis reference RNA (Figures S2–S4), indicating that the NOA3 subclass has a mild defect in spermatogenesis. This notion is supported by the fact that the expression of *INHBB* encoding inhibin β subunit B in the NOA3 subclass is normal while NOA1 and NOA2 subclasses showed low levels, indicating that inhibin β may be a marker of testicular dysfunction, as previously reported [18].

### Verification of Between-Subclass Differences in Testicular Expression by Quantitative Real-Time RT-PCR

To evaluate the appropriateness of microarray data on transcripts representing expression differences between NOA subclasses, we selected 53 with high significance (*p* < 0.01, Tukey's *post hoc* test, Figure S1 and Table S1) out of the 149 differentially expressed transcripts and subjected them to real-time RT-PCR analysis. Of the 53 transcripts, the highly homologous VCX family genes, *VCX*, *VCX2*, and *VCX3A*, were detected with non-specific assay as a mixture of transcripts. Thus, 50 genes and one gene mixture were subjected to real-time RT-PCR. As shown in Figure S5, real-time RT-PCR data of the 51 transcripts were highly correlated with the results of microarray analysis, the squares of correlation coefficients (*R* ^2^) ranging from 0.40 (CT45–2) to 0.90 (GAJ). This validation analysis also provided statistically positive evidence on between-subclass differences for all of the 51 transcripts (*p* < 0.05 with Kruskal-Wallis test, data not shown).

### Screening of Candidate Genes for Genetic Susceptibility for NOA

One approach to prioritizing candidate genes for genetic susceptibility underlying NOA is to adopt gene expression data from NOA tissues. Genes that show differences in expression level between NOA subclasses regardless of biological impact were selected based on the concept that polymorphic variation in gene expression among unrelated individuals is largely due to polymorphisms in DNA sequence [19,20]. 52 genes having statistical differences in expression (*p* < 0.01, Table S1) were regarded as candidates for allelic association with NOA. Despite the fact that these genes were not selected based on pathological relevance to NOA, genes such as *SYCP3*, *DAZL*, and *INHBB*, which were reported to function in spermatogenesis were included [21–23]. 191 single nucleotide polymorphisms (SNPs) of 42 genes were subjected to allelic association study with 190 NOA patients and 190 fertile men in the first round of screening. Ten genes (*CTAG1B*, *LOC158812*, *LOC255313, MAGEA2*, *PEPP-2*, *TSPY1*, *TSPY2*, *VCX3A*, *VCY*, and *XAGE1*) were not analyzed because no gene-based SNPs with minor allele frequency (MAF) > 0.05 could be found. We identified seven genes (*ART3*, *LOC92196*, *NYD-SP20*, *PAGE5*, *TEX14*, *TKTL1*, and *XAGE5*) with at least one SNP showing a discrepancy in MAF of 5% or greater between cases and controls (Table S2). Forty-four SNPs in the seven genes were subjected to a second round of screening by increasing sample size (380 NOA patients and 380 fertile men). After the two rounds of screenings, only one SNP (rs6836703) of *ART3* (ADP-ribosyltransferase 3) was positively associated with NOA after Bonferroni's correction for multiple testing (Table 3; χ^2^ = 11.7, corrected *p* = 0.027).

**Table 3:**
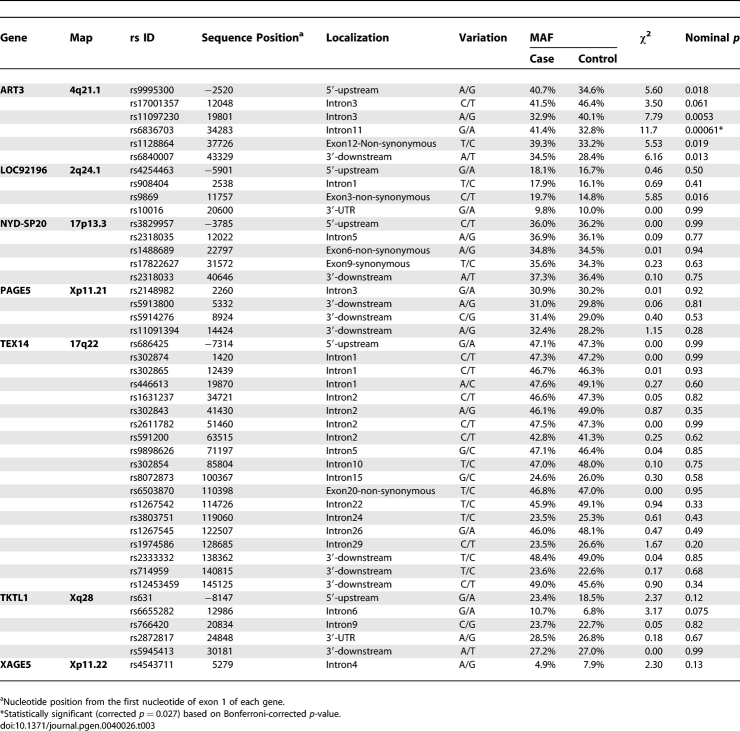
Analyses of Allelic Association between 44 SNPs of Seven Candidate Genes and Japanese NOA in Second Round of Initial Screening (380 Cases versus 380 Controls)

### Allelic Association Study with *ART3*

We focused on *ART3* based on the result of the two rounds of screenings, and identified 38 SNPs with MAF > 0.1 by database search or direct sequencing of the gene. 442 NOA patients (cases) and 475 fertile men (controls) were genotyped. Because we intended in this study to find a common genetic cause for NOA, patients with microdeletions of the Y chromosome at the azoospermia factor (AZF) locus, one of the major causes of NOA [1,2], were not excluded from the cases. However, to characterize the cases in regard to the AZF deletions, we examined the incidence of the deletions in a subset of the cases. Of the 442 NOA patients, 99 were examined by PCR-based screening. Fourteen (14.1 %) of the 99 cases examined showed the AZF deletions, and NOA patients with AZFc deletions were most frequent among the 14 cases (data not shown). The overall deletion frequency was comparable to those of other studies [1,2], in which the higher incidence of AZFc deletions also was observed. The clinical characteristics of patients with the AZF deletions did not differ from those of the other NOA patients (data not shown).

**Figure 5:**
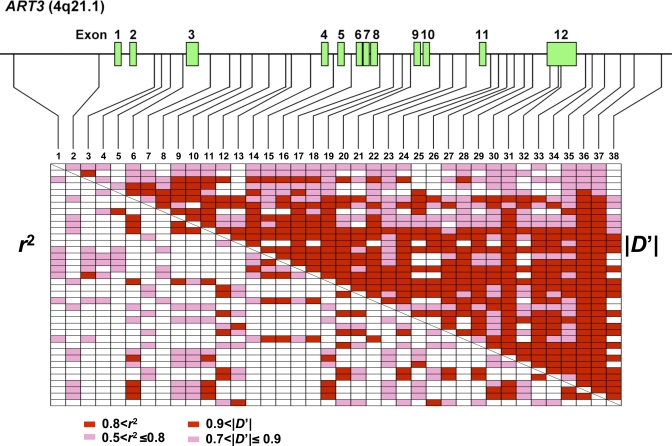
Linkage Disequilibrium Pattern of *ART3* The gene structure together with the position of 38 SNPs is shown. Pairwise LD coefficients, *D*' and *r* ^2^, of controls were determined and expressed as a block structure. In the schematic block, red boxes indicate pairwise LD of |*D*'| > 0.9 or *r* ^2^ > 0.8 and pink boxes 0.9 ≥ |D'| > 0.7 or 0.8 ≥ *r* ^2^ > 0.5. Blank boxes represent |*D*'| ≤ 0.7 or *r* ^2^ ≤ 0.5.

Linkage disequilibrium map showed that all of the SNPs of *ART3* were in near complete LD evaluated with *D*' statistic (|*D*'| > 0.7) in both cases and controls (only controls are displayed in Figure 5). None of the SNPs in the controls showed deviation from Hardy-Weinberg's equilibrium at a threshold of *p* < 0.01 (data not shown). As shown in Table 4, SNPs showing positive associations based on nominal *p*-values were widely distributed throughout *ART3*. The most significant association was observed with ART3-SNP25 (rs6836703) located in intron 11 of *ART3* (χ^2^ = 9.16, nominal *p* = 0.0025, odds ratio [95% CI] = 1.34 [1.11–1.63]). We applied the permutation method for adjustment of multiple testing to avoid a false positive result [24]. A total of four SNPs including ART3-SNP25 met the empirical significance level of *p* < 0.05 (Table 4).

**Table 4:**
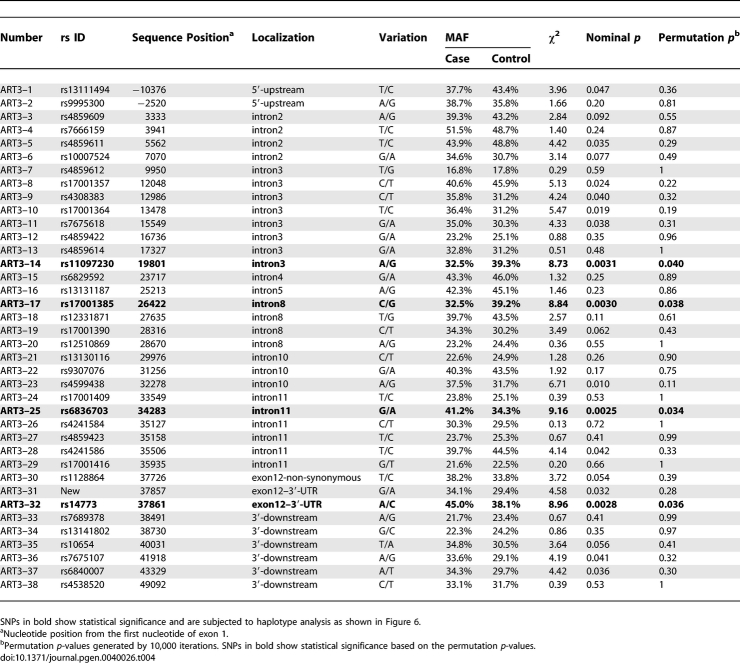
Allelic Association of 38 SNPs in *ART3* with NOA in Japanese Population (442 NOA Patients versus 475 Controls)

**Figure 6:**
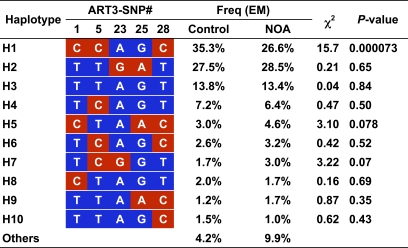
Haplotype-based Association Study of *ART3* The expectation-maximization (EM) algorithm [37] was used to infer *ART3* haplotype frequencies with genotyping data of five tag SNPs, ART3-SNP1, 5, 8, 23, 25, and 28 (see Table 4). At the respective SNP sites, red and blue boxes represent minor and major alleles, respectively.

For the haplotype-based association study, we first selected five SNPs (ART3-SNP1, 5, 23, 25, and 28) as tag SNPs captured through LD in *ART3* from 15 SNPs with nominal *p* < 0.05 at a threshold of *r* ^2^ ≥ 0.8 with Tagger software [25]. Haplotype frequencies were inferred using an expectation-maximization (EM) algorithm. After excluding rare haplotypes (frequency < 0.01), association of ART3 haplotypes with NOA was examined in 442 cases and 475 controls. Haplotype H1, the most common haplotype in controls, was under-represented in cases with significance (Figure 6; 26.6% in cases and 35.3 % in controls; χ^ 2^ = 15.7, df = 1, nominal *p* = 0.000073), indicating a protective impact of haplotype H1. After Bonferroni's correction for multiple testing, a protective effect of haplotype H1 was still significant (corrected *p* = 0.00080). Other haplotypes showed no significant difference in frequencies between cases and controls (Figure 6). We also applied a Bayesian algorithm for phasing haplotypes with PHASE version 2.1.1 [26,27]. Regardless of haplotype-phasing methods, haplotype H1 was the most frequent in controls (26.4% in cases and 35.0% in controls), and a significant difference in haplotype H1 frequency between cases and controls was observed (permutation *p* < 0.0001 in global comparison, generated after 10,000 iterations).

### Clinical Relevance of the Haplotype Associations

**Figure 7:**
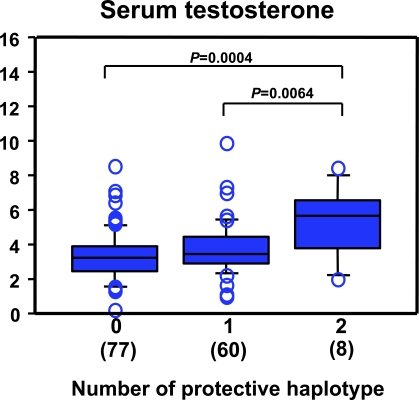
Diplotype-Specific Differences in Serum Testosterone Levels in NOA Patients Three diplo-groups (code 0, 1, and 2) were defined by the number of *ART3* haplotype H1 carried. The serum testosterone levels were significantly different among the three groups (Kruskal-Wallis; df = 2, *p* = 0.0093) by Bonferroni/Dunn *post hoc* test.

The functional relevance of haplotype H1 in comparison with the clinical data was then explored. Diplotype was inferred with EM algorithm, and three categories (code 0, 1, and 2) were defined by the number of haplotype H1 carried without counting the other haplotypes, and nonparametric analysis of variance test with clinical data was performed. Serum levels of hormones (LH, FSH, and testosterone), other biochemical and pathophysiological markers, and Johnsen's score were analyzed by Kruskal-Wallis test with a Bonferroni/Dunn *post hoc* test between the three diplo-groups. Serum testosterone levels were significantly different among the three groups (Figure 7; df = 2, *p* = 0.0093), but there were no significant differences in other clinical markers. Post hoc pairwise comparisons revealed that serum testosterone levels were significantly higher in the subgroup having two copies of haplotype H1 than in a subgroup with one or no haplotype H1 (*p* = 0.0064 or *p* = 0.0004, respectively, Figure 7). PHASE-inferred individual diplotypes also revealed a similar correlation between diplo-groups of haplotype H1 and serum testosterone levels (data not shown).

### ART3 Protein Localization in Azoospermic Testis

**Figure 8:**
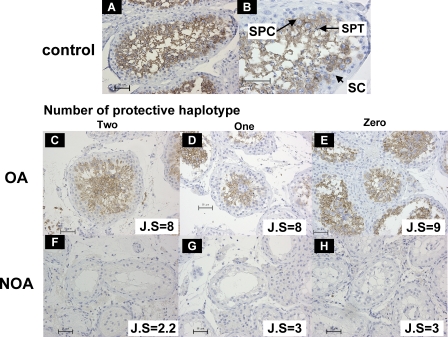
Immunohistochemical Analysis of ART3 Expression in Human Testes Representative seminiferous tubules in testicular sections from normal controls (A, B), OA (C–E), and NOA patients (F–H) are shown. Arrows indicate spermatocytes (SPC); spermatids (SPT); and Sertoli cells (SC) (B). ART3 protein was immunostained with anti-ART3 antibody; ART3-positive spermatocytes (SPC) are noted as brown staining cells (A–E). No marked differences in testicular ART3 expression among the three ART3 diplo-groups carrying none, one, or two copies of the protective haplotype H1were observed in OA (C–E) and NOA (F–H) patients. Magnification is 60× except in (B) (120×).

ART3 protein expression in azoospermic testes was examined by immunohistochemical analysis. As shown in Figure 8, specific staining of ART3 protein was predominantly observed in spermatocytes in OA testes (Figure 8C–8E) as well as in normal testes from individuals of accidental sudden-death (Figure 8A and 8B). Staining was not observed in other stages of undifferentiated germ cells or Sertoli cells in the seminiferous tubules, or the interstitial tissues such as Leydig cells. On the other hand, we did not detect any ART3 protein in NOA testes with Johnsen's scores ranging from 2 to 3, which showed no spermatocytes, spermatids, or spermatozoa in the seminiferous tubules (*n* = 12 samples; Figure 8F–8H). There was no marked difference in testicular ART3 protein expression among the three *ART3* diplo-groups carrying none, one, or two copies of haplotype H1.

## Discussion

### Genomic Analysis of NOA

Our investigation was designed to clarify the pathogenesis of NOA using global gene expression analyses of testis samples from NOA patients and to identify genetic susceptibilities underlying NOA from the genes differentially expressed. Large families with multiple generations having NOA cannot be expected due to the nature of infertility, so linkage study is impractical for NOA and has not been reported. Alternatively, allelic association study is a practical approach to identification of genetic susceptibility underlying NOA. Thus far, more than 80 genes have been identified as essential for male infertility in humans and mice [3]. Genes on the Y chromosome were emphasized because of observed microdeletions in patients, and genes such as DAZ and HSFY were examined for possible susceptibility genes [28,29]. Recently, homozygous mutation of the aurora kinase C gene was identified in large-headed multiflagellar polyploid spermatozoa, a rare form of infertility, using homozygosity mapping [30]. In the current study, we applied a novel approach to identify common susceptibility genes for NOA by applying global gene expression analysis of NOA testes. Based on the hypothesis that a common variant of a susceptibility gene has resulted in altered expression in tissues relevant to disease etiology [31], we first elucidated the gene expression profile in testes of NOA patients and characterized the genetic pathways that were either under-expressed or over-expressed. Because spermatogenesis is a complex differentiation process, NOA could result from a defect at any stage of the process. Thus, gene expression profiling of NOA tissues might well be confounded by the difficulty of discerning the differential stage and the pathological status. Feig et al. [4] examined stage-specific gene expression profile**s** in human NOA patients after classification on the basis of Johnsen's score. The testis tissues were classified into four groups showing Sertoli-cell only syndrome, meiotic arrest, testicular hypospermia, and testicular normospermia, corresponding to Johnsen's score 2, 5, 8, and 10, respectively, and stage-specific differential gene expression was monitored. We sought to identify susceptibility genes underlying NOA that could affect any stage of spermatogenesis. Testis samples subgrouped according to Johnsen's score in advance might identify genes affecting multiple stages of spermatogenesis. Therefore, we globally subgrouped the samples at diverse stages of differentiation using an NMF method for reducing multidimensionality that is appropriate for application to high dimensional biological data. The NMF method subgrouped three classes, NOA1, NOA2, and NOA3, which also were unequivocally subgrouped by the HC approach (Figure 3). Notably, NOA1 and NOA2 represent a pathologically similar type showing low Johnsen's score, but were subclassified because of their distinct gene expression pattern. NOA1 and NOA2 showed differences in LH, FSH, and testosterone levels, thus establishing meaningful biological significance of the sub-classes (Table 2).

### Genetic Susceptibility to NOA

In the current study, we adopted a novel approach to select candidate susceptibility genes for NOA. Global gene expression analyses were performed on NOA testes, and 52 genes were selected according to differential gene expression between NOA subclasses with a strict statistical criterion (*p* < 0.01 with Tukey's *post hoc* test). Despite the fact that our selection criteria relied only on data regarding differences in gene expression and did not include any biological assumptions, many of the genes were related to spermatogenesis based on Gene Ontology analyses (Figure 4; Table 1). 191 SNPs of 42 genes were screened, and only one gene, *ART3*, showed a positive association after the two rounds of screening. Multiple SNPs of *ART3* were significantly associated with NOA, the most significant association being observed with ART3-SNP25 (rs6836703, nominal *p* = 0.0025, permutation *p* = 0.034; Table 4). We also detected a protective haplotype, H1, which was the most common form and was strongly associated with NOA (nominal *p* = 0.000073, corrected *p* = 0.00080, Figure 6). In addition, diplotype analysis showed that individuals carrying at least one haplotype H1 showed an elevated plasma testosterone level (Figure 7).

### Functional Relevance of ART3 in the Pathogenesis of NOA

*ART3* is a member of the mono-ADP-ribosyltransferase family genes. The biological function of ART3 remains obscure, as ART3 does not display any detectable arginine-specific transferase activity due to lack of the active site motif (R-S-EXE) that is essential for catalytic activity. Since differentiation of stage-specific expression of ART3 in testis has been reported, protein expression being exclusively present in spermatocytes but absent in spermatozoa [32], a genetic variation of *ART3* might well lead to a functional defect in the process of spermatogenesis. Haplotype H1 of *ART3*, comprising all of the disease-protective alleles at the respective SNP sites, was under-represented in the patients. However, functional disturbance associated with haplotype H1 is so far undetermined despite the fact that several experiments designed to demonstrate haplotype-specific differences in expression level have been performed. Thus, it is possible that this haplotype represents fine tuning that maintains normal maturation of spermatocytes and improves the efficiency of spermatogenesis.

In conclusion, genome-wide gene expression analyses identified differentially expressed genes of NOA subclasses, and *ART3* was identified as a susceptibility gene underlying NOA. This genetic study constitutes only first-stage evidence of association because only Japanese individuals were included, so further replication in independent case-control samples is required to confirm the role of the *ART3* haplotype in genetic risk for NOA. Although further functional evidence is also required, these results provide insight into the pathoetiology of NOA as well as reproductive fitness at the molecular level, and suggest a target for therapy.

## Materials and Methods

### Participants.

Testicular biopsy specimens for microarray analysis were obtained from 47 Japanese patients (aged from 24 to 52 years) with NOA and 11 (aged from 22 to 57 years) with OA, each of whom also underwent testicular sperm extraction (TESE) for assisted reproduction and/or diagnostic biopsy for histological examination. The biopsies for microarray analysis and histological examination were mainly sampled from unilateral, multiple testicular sites in the respective patients. Each patient was first assigned to azoospermia by showing no ejaculated spermatozoa in a semen examination. Subsequently, OA was defined as follows: (1) motile spermatozoa were sampled from microsurgical epididymal sperm aspiration (MESA), or (2) a considerable number of mature spermatozoa was sampled from TESE. NOA was tentatively defined as having no epididymal and/or testicular spermatozoa. The degree of spermatogenic defect was histologically evaluated according to Johnsen's score [11]. At least three biopsies from the same individual were taken, and the average Johnsen's scores in the NOA and OA groups ranged from 1 to 6.5 and from 5.1 to 9, respectively. In most patients, preoperative levels of serum follicle-stimulating hormone (FSH), leutenizing hormone (LH), and total testosterone were measured. The infertile male patients who visited Niigata University, Tachikawa Hospital, and St. Mother's Hospital received a routine semen examination according to 1999 WHO criteria. Based on this analysis, sperm were counted and the patients who had no ejaculated sperms were enrolled for a case-control association study. In total, 442 patients were ascertained to have NOA. In the current study, azoospermia patients with varicocele, ejaculatory dysfunction, endocrinopathy, or histologically examined OA as defined above were excluded. 475 fertile men having no specific clinical record were recruited in Niigata University. The ethics committees of Niigata University, Tachikawa Hospital, St. Mother's Hospital, and Tokai University approved the study protocols, and each participant gave written informed consent. Genomic DNA was prepared from blood white cells by Dneasy (Qiagen, Tokyo, Japan) or salivas by phenol/chloroform extraction.

To examine microdeletion of the Y chromosome in a subset of NOA patients, PCR-based diagnostic technique was used as follows: PCR amplifications with fluorescence (FAM or HEX)-labeled primers were performed to obtain fragments encompassing each of 13 STS markers in and around azoospermia factor (AZF) regions of the Y chromosome (in AZFa: SY83, SY95 and SY105; in AZFb: SY118, G65320, SY126 and SY136; in AZFc: SY148, SY149, SY152, SY283 and SY1291; in the heterochromatin distal to AZFc: SY166). Primer sequences and PCR conditions are available from the authors on request. PCR-amplified fragments were run on the ABI PRISM 3100 Genetic Analyzer (Applied Biosystems, Tokyo, Japan), and Y-chromosome microdeletion was determined with GENESCAN software (Applied Biosystems).

### Microarray analysis of testis samples.

Total RNA from testicular biopsy was extracted using TRIzol reagent (Invitrogen, Carlsbad, CA, USA) and quantity and quality of the extracted RNA were examined with 2100 Bioanalyzer (Agilent Technologies, Palo Alto, CA, USA) using RNA 6000 Nano LabChip (Agilent Technologies). Human Testis Total RNA (BD Biosciences, San Jose, CA, USA), a histologically normal testicular RNA pooled from 39 Caucasians, was used as a common reference in two-color microarray experiments.

For fluorescent cRNA synthesis, high-quality total RNA (150 ng) was labeled with the Low RNA Input Fluorescent Linear Amplification Kit (Agilent Technologies) according to the manufacturer's instructions. In this procedure, cyanine 5-CTP (Cy5) and cyanine 3-CTP (Cy3) (PerkinElmer, Boston, MA, USA) were used to generate labeled cRNA from the extracted patient RNA and the reference RNA, respectively. Labeled cRNAs (0.75 μg each) from one patient and the common reference were combined and fragmented in a hybridization mixture with the *In Situ* Hybridization Kit Plus (Agilent Technologies). The mixture was hybridized for 17 hours at 65°C to the Agilent Human 1A(v2) Oligo Microarray, which carries 60-mer probes to 18,716 human transcripts. After hybridization, the microarray was washed with SSC buffer, and then scanned in Cy3 and Cy5 channels with the Agilent DNA Microarray Scanner model G2565AA (Agilent Technologies). Signal intensity per spot was generated from the scanned image with Feature Extraction Software ver7.5 (Agilent Technologies) in default setting. Spots that did not pass quality control procedures were flagged and removed for further analysis.

The Lowess (locally weighted linear regression curve fit) method was applied to normalize the ratio (Cy5/Cy3) of the signal intensities generated in each microarray with GeneSpring GX 7.3 (Agilent Technologies). Compared with the expression level of reference RNA, the NOA group, with expression undergoing a 2-fold mean change or more was extracted; the OA group comprised transcripts showing less than 2-fold mean expression change (Figure 1A). Of the transcripts included in both groups, only those with a statistically significant difference in expression between NOA and OA testes (based on lowess-normalized natural log[Cy5/Cy3], Bonferroni's corrected *p* < 0.05) were counted as NOA-related target genes. To elucidate the molecular subtypes of NOA, we adopted the non-negative matrix factorization (NMF) algorithm, which has been recently introduced to analysis of gene expression data [5,6]. For this analysis, a complete dataset without missing values was generated from raw values of Cy5 intensities for the NOA-related target genes in the NOA samples, and used to clarify NOA heterogeneity using three M-files (available from the following URL; http://www.broad.mit.edu/cgi-bin/cancer/publications/pub_paper.cgi?mode=view&paper_id=89) for MATLAB (Mathworks, Natick, MA, USA). According to the subclassification of NOA samples, transcripts differentially expressed between NOA subclasses were determined by one-way ANOVA, followed by Tukey's *post hoc* test in GeneSpring GX. For multiple test corrections in this statistical analysis, we used the Benjamini-Hochberg procedure [33] of controlling the false discovery rate (FDR) at the level of 0.05 or 0.01. To analyze which categories of Gene Ontology were statistically overrepresented among the gene lists obtained, we used GO Browser, an optional tool in GeneSpring GX, where the statistical significance was determined by Fisher's exact test. The microarray data reported in this paper have been deposited in the Gene Expression Omnibus (GEO, http://www.ncbi.nlm.nih.gov/geo/) database, and are accessible through GEO Series accession number GSE9210.

### Quantitative real-time RT-PCR analysis for validation of between-subclass differences in gene expression.

Quantitative real-time RT-PCR analysis was used to verify the microarray data on 53 transcripts representing differential expressions between NOA subclasses with high significance (*p* < 0.01). Among 53 transcripts, VCX (NM_013452), VCX2 (NM_016378), and VCX3A (NM_016379) were examined as a single transcript because sequence homologies between the three transcripts prevented development of appropriate assays for discrimination. Testicular total RNA (1 μg) subjected to microarray analysis was used as a template in first-strand cDNA synthesis with SuperScript III First-Strand Synthesis System (Invitrogen). Each single-stranded cDNA was diluted one-tenth for a subsequent real-time RT-PCR using SYBR *Premix Ex Taq* (Perfect Real Time) (TAKARA BIO, Otsu, Japan) on the ABI PRISM 7900HT Sequence Detection System (Applied Biosystems) according to the manufacturer's instructions. The PCR primers for 43 transcripts showing between-subclass differences with high significance and *GAPDH* were designed and synthesized by TAKARA BIO Inc., or QIAGEN GmbH (as the QuantiTect Primer Assay). In the real-time RT-PCR analysis for the nine remaining transcripts, we used TaqMan Gene Expression Assays (Applied Biosystems) with TaqMan Universal PCR Master Mix (No AmpErase UNG version) according to the manufacturer's instructions (Applied Biosystems). The detailed information on the primer sequences used and/or the assay system selected are summarized in Table S3. A relative quantification method [34] was used to measure the amounts of the respective genes in NOA testes, normalized to *GAPDH* as an endogenous control, and relative to Human Testis Total RNA (BD Biosciences) as a reference RNA. Statistical significance between NOA subclasses was determined by Kruskal-Wallis test, followed by multiple comparisons; *p* < 0.05 was considered significant.

### SNP selection of candidate genes for NOA and genotyping.

Based on gene expression data of NOA testes, we selected 52 genes (encoding 53 transcripts) as candidates for genetic susceptibilities underlying NOA. SNPs of the candidate genes with minor allele frequency (MAF) > 0.05 were obtained from the NCBI dbSNP database (http://www.ncbi.nlm.nih.gov/SNP/), and applied to an initial screening. Of the 52 candidate genes, 10 genes (CTAG1B, LOC158812, LOC255313, MAGEA2, PEPP-2, TSPY1, TSPY2, VCX3A, VCY, and XAGE1) were excluded from the initial screening because gene-based SNPs with MAF > 0.05 were not found in the public SNP database. A total of 191 SNPs of 42 genes were genotyped in the screening with TaqMan SNP Genotyping Assays on the ABI PRISM 7900HT Sequence Detection System (Applied Biosystems). 190 NOA patients (cases) and 190 fertile men (controls) were genotyped in the first round screening. For genes with at least one SNP showing a discrepancy in MAF of 5% or greater between cases and controls, the sample size was increased to 380 cases and 380 controls in the second round.

After two rounds of initial screening, additional SNPs of *ART3* were selected from dbSNP or identified by direct sequencing of all 12 exons of the gene (Ensemble transcript ID ENST00000355810) and splice acceptor and donor sites in the intron using the genomic DNAs from 95 infertile patients as PCR templates. A total of 38 SNPs of *ART3* were finally genotyped on 442 cases and 475 controls by TaqMan SNP Genotyping Assays or by direct sequencing with BigDye Terminators v3.1 Cycle Sequencing Kit (Applied Biosystems) on ABI PRISM 3700 DNA analyzer.

### Statistical analyses in association study.

Pairwise linkage disequilibrium (LD), using the standard definition of *D*' and *r* ^2^ [35,36], was measured with SNPAlyze v5.0 software (DYNACOM, Mobara, Japan). To construct *ART3* haplotypes in phase-unknown samples, tag SNPs of *ART3* were selected with Tagger software [25], incorporated in the Haploview. The expectation-maximization (EM) algorithm [37] and PHASE version 2.1.1 [26,27] was used to infer haplotype frequencies and individual diplotypes for *ART3*. Differences in allelic and haplotype frequencies were evaluated using a case-control design with the chi-square test. For an adjustment of multiple testing, we applied a permutation method with Haploview version 3.32 software, or Bonferroni's method to determine corrected *p*-values.

To investigate association of the *ART3* diplotype with clinical phenotypes such as serum hormone levels, differences among the three categories (code 0, 1, and 2), defined by the number of the most significant haplotype, were statistically examined by Kruskal-Wallis test, followed by Bonferroni/Dunn *post hoc* test (StatView version 5.0, SAS Institute, Cary, NC, USA).

### Immunohistochemistry.

To examine cellular localization of ART3 protein in azoospermic testes, testicular biopsy specimens from 15 OA and 12 NOA patients were subjected to immunohistochemistry. Four postmortem testicular tissues of accidental sudden-deaths were used as normal controls. The testicular tissues were fixed in 10% buffered formalin and embedded in paraffin. Cryosections (3 μm thickness) were pre-incubated with the Histofine Antigen-Retrieval Solution (1:10 dilution; Nichirei Bioscience, Tokyo, Japan) for 10 minutes at 95 °C. The sections were then incubated with primary ART3 antibody (1:4,000; Abnova, Taipei, Taiwan), then with IgG2b isotype (1:4,000; MBL International, Woburn, USA) for 60 minutes at room temperature. After washing with PBS, the sections were incubated with the Histofine Simple Stain Max-PO (Multi) (1:5 dilution; Nichirei Bioscience) for 30 minutes at room temperature, and then reacted with DAB (Nichirei Bioscience) for 10 minutes at room temperature. Haematoxylin was used for counterstaining.

## Supporting Information

Figure S1 Statistical Analysis Reveals Transcripts Differentially Expressed among Three NOA Subclasses Venn diagram summaries show the number of transcripts differentially expressed with significance by Tukey's *post hoc* test in each comparison (see Table S1)
(694 KB EPS)

Figure S2 Comparisons of Expression Levels of 149 Transcripts Expressed Differentially between Three NOA Subclasses in Microarray Analysis (Part I) Natural log-transformed normalized ratios of NOA to testis reference (*y*-axes) were subjected to statistical analysis, as described in Materials and Methods. Each column represents mean ± standard error of the mean. The 53 transcripts with highly significant (*p* < 0.01, Tukey test) differences between the three NOA subclasses are shown in red.
(773 KB EPS)

Figure S3 Comparisons of Expression Levels of 149 Transcripts Expressed Differentially between Three NOA Subclasses in Microarray Analysis (Part II) Natural log-transformed normalized ratios of NOA to testis reference (*y*-axes) were subjected to statistical analysis, as described in Materials and Methods. Each column represents mean ± standard error of the mean. The 53 transcripts with highly significant (*p* < 0.01, Tukey test) differences between the three NOA subclasses are shown in red.
(798 KB EPS)

Figure S4 Comparisons of Expression Levels of 149 Transcripts Expressed Differentially between Three NOA Subclasses in Microarray Analysis (Part III) Natural log-transformed normalized ratios of NOA to testis reference (*y*-axes) were subjected to statistical analysis, as described in Materials and Methods. Each column represents mean ± standard error of the mean. The 53 transcripts with highly significant (*p* < 0.01, Tukey test) differences between the three NOA subclasses are shown in red.
(822 KB EPS)

Figure S5 Correlations of Testicular Gene Expression Evaluated by Microarray and Quantitative Real-Time RT-PCR Analyses Expression levels of 51 transcripts with highly significant (*p* < 0.01) differences in expression among the three NOA subclasses were quantified by real-time RT-PCR method as described in Material and Methods. Squares of correlation coefficients (*R* ^2^) for the respective transcripts were calculated between normalized expression ratios of NOA to testis reference in microarray data (*x*-axes) and the corresponding ratios obtained by real-time RT-PCR analysis (*y*-axes)
(1.7 MB EPS)

Table S1 149 Transcripts Representing Statistically Significant (*p* < 0.05) Differences in Testicular Expression between Three NOA Subclasses (68 KB XLS)

Table S2 Comparison of Minor Allele Frequencies (MAFs) at 191 SNPs of 42 Genes between 190 Infertile Patients and 190 Fertile Males (First Round of Initial Screening) (415 KB DOC)

Table S3 Quantitative Real-time RT-PCR Assays for 51 Transcripts Representing Highly Significant Differences between NOA Subclasses (30 KB XLS)

## Abbreviations

ART3: ADP-ribosyltransferase 3
AZF: azoospermia factor
coph: cophenetic correlation coefficient
Cy3: cyanine 3-CTP
Cy5: cyanine 5-CTP
EM: expectation–maximization
FDR: false discovery rate
FSH: follicle-stimulating hormone
GO: Gene Ontology
HC: hierarchical clustering
LD: linkage disequilibrium
LH: leutenizing hormone
MAF: minor allele frequency
MESA: microsurgical epididymal sperm aspiration
NMF: non-negative matrix factorization
NOA: non-obstructive azoospermia
OA: obstructive azoospermia
*R*^2^: square of correlation coefficient
SNP: single nucleotide polymorphism
TESE: testicular sperm extraction
