# Supplementary material for: Genome-Wide Expression of Azoospermia Testes Demonstrates a Specific Profile and Implicates ART3 in Genetic Susceptibility
Source: PLoS Genet. 2008 Feb 8;4(2):e26. doi: 10.1371/journal.pgen.0040026 (PMC2233677; doi:10.1371/journal.pgen.0040026)
Supplement: Supplementary file 1 [file pgen.0040026.st002.doc]

**Table S2. Comparison of minor allele frequencies (MAFs) at 191 SNPs of 42 genes between 190 infertile patients and 190 fertile males (1st round of initial screening)**

| Gene | rs ID | Sequence Position^a^ | Localization | Variation | MAF | |
| --- | --- | --- | --- | --- | --- | --- |
| (Map) |  |  |  |  | Case | Control |
| ACAT2 | rs2146162 | -1984 | 5'-upstream | G/A | 39.1% | 35.8% |
| (6q25.3) | rs9364535 | 7532 | intron4 | A/T | 39.0% | 35.6% |
|  | rs2273828 | 23963 | 3'-downstream | A/G | 19.5% | 19.7% |
|  |  |  |  |  |  |  |
| **ART3** | rs9995300 | -2520 | 5'-upstream | A/G | 41.3% | 36.7% |
| (4q21.1) | rs17001357 | 12048 | intron3 | C/T | 40.5% | 42.8% |
|  | rs11097230 | 19801 | intron3 | A/G | 34.0% | 36.8% |
|  | **rs6836703** | 34283 | intron11 | G/A | **42.9%** | **36.6%** |
|  | rs1128864 | 37726 | exon12-non-synonymous | T/C | 39.5% | 36.1% |
|  | rs6840007 | 43329 | 3'-downstream | A/T | 34.7% | 31.9% |
|  |  |  |  |  |  |  |
| BUB1 | rs13415981 | -3947 | 5'-upstream | A/G | 6.7% | 7.3% |
| (2q14) | rs11903773 | 9926 | intron6 | C/T | 7.2% | 6.7% |
|  | rs7609252 | 16200 | intron9 | A/G | 6.8% | 7.6% |
|  | rs7581640 | 34693 | intron19 | T/C | 6.0% | 5.4% |
|  | rs13398617 | 37155 | intron24 | T/C | 6.9% | 7.4% |
|  | rs13419537 | 43371 | 3'-downstream | C/T | 6.8% | 8.0% |
|  |  |  |  |  |  |  |
| CDCA2 | rs7819740 | -4478 | 5'-upstream | G/T | 33.9% | 35.2% |
| (8p21.2) | rs3812422 | 149 | 5’-UTR | A/C | 31.8% | 32.5% |
|  | rs4872315 | 16088 | intron7 | T/G | 33.4% | 34.4% |
|  | rs11135869 | 18842 | intron7 | G/A | 33.4% | 34.3% |
|  | rs2271104 | 27124 | intron11 | C/T | 33.4% | 33.9% |
|  | rs7826705 | 36321 | intron13 | T/A | 30.1% | 34.1% |
|  | rs4872318 | 47819 | exon15-non-synonymous | G/A | 16.8% | 16.2% |
|  |  |  |  |  |  |  |
| CDCA5 | rs7943007 | -5574 | 5'-upstream | C/G | 13.0% | 13.4% |
| (11q12.1) | rs489489 | 6086 | 3’-UTR | C/A | 13.2% | 13.6% |
|  |  |  |  |  |  |  |
| CDT1 | rs565211 | -1935 | 5'-upstream | C/T | 25.1% | 29.2% |
| (16q24.3) | rs475667 | 780 | exon2-synonymous | T/C | 19.6% | 19.5% |
|  | rs480727 | 2042 | exon3-non-synonymous | A/G | 24.2% | 26.8% |
|  | rs8191478 | 7242 | 3'-downstream | C/T | 43.3% | 41.4% |
|  |  |  |  |  |  |  |
| CT45-2 | rs9633172 | 11971 | 3'-downstream | A/G | 11.6% | 8.2% |
| (Xq26.3) |  |  |  |  |  |  |
|  |  |  |  |  |  |  |
| DAZL | rs13064112 | -5891 | 5'-upstream | A/G | 29.7% | 29.5% |
| (3p24.3) | rs4234538 | -542 | 5'-upstream | C/G | 23.4% | 28.2% |
|  | rs11710967 | 6931 | exon2-synonymous | G/A | 6.8% | 6.5% |
|  | rs3773826 | 12693 | intron9 | G/A | 10.5% | 11.1% |
|  | rs10510452 | 21958 | 3'-downstream | A/G | 15.6% | 15.8% |
|  |  |  |  |  |  |  |
| DDX4 | rs16884506 | -6616 | 5'-upstream | A/G | 31.2% | 27.7% |
| (5q11.2) | rs3761772 | 235 | intron1 | A/G | 31.8% | 30.0% |
|  | rs17699426 | 4634 | intron1 | A/G | 12.4% | 13.5% |
|  | rs12653775 | 12368 | intron2 | C/T | 43.8% | 43.3% |
|  | rs4865627 | 17485 | intron2 | C/G | 48.3% | 43.5% |
|  | rs3761776 | 25139 | intron3 | C/A | 45.3% | 42.4% |
|  | rs12652234 | 29062 | intron5 | C/G | 43.7% | 39.1% |
|  | rs7378817 | 36492 | intron6 | T/C | 28.9% | 27.3% |
|  | rs3789195 | 60193 | intron16 | T/C | 47.1% | 43.7% |

Table S2. Continued

| Gene | rs ID | Sequence Position^a^ | Localization | Variation | MAF | |
| --- | --- | --- | --- | --- | --- | --- |
| (Map) |  |  |  |  | Case | Control |
| (DDX4) | rs2619046 | 63681 | intron17 | G/A | 47.6% | 44.4% |
|  | rs12518406 | 71863 | intron17 | A/C | 31.3% | 27.5% |
|  | rs3815767 | 77495 | intron20 | G/A | 31.3% | 28.0% |
|  | rs162089 | 84822 | 3'-downstream | A/G | 43.4% | 39.3% |
| DDX43 | rs558198 | 85 | 5'-UTR | G/C | 23.7% | 24.7% |
| (6q12-q13) | rs3757106 | 5740 | intron3 | T/G | 11.3% | 9.4% |
|  | rs3734516 | 10686 | intron5 | C/T | 20.5% | 19.1% |
|  | rs9442907 | 18891 | intron11 | G/A | 13.5% | 11.7% |
|  | rs311686 | 21402 | exon15-non-synonymous | C/T | 46.6% | 47.3% |
|  | rs4032696 | 29240 | 3'-downstream | T/A | 25.8% | 26.0% |
|  |  |  |  |  |  |  |
| DMRTB1 | rs7543581 | -2918 | 5'-upstream | C/T | 44.4% | 40.7% |
| (1p32.3) | rs1288628 | 964 | intron1 | A/C | 37.5% | 38.4% |
|  | rs1296439 | 13463 | 3'-downstream | T/G | 48.1% | 48.6% |
|  |  |  |  |  |  |  |
| DMRTC2 | rs4444435 | -5499 | 5'-upstream | T/G | 44.4% | 43.3% |
| (19q13.2) | rs2305809 | 5604 | 3’-UTR | G/A | 44.7% | 43.8% |
|  | rs6509002 | 9044 | 3'-downstream | C/T | 44.0% | 45.1% |
|  |  |  |  |  |  |  |
| DSCR8 | rs9980291 | -8010 | 5'-upstream | C/T | 15.3% | 12.5% |
| (21q22.2) | rs2211855 | 1252 | intron2 | G/A | 14.1% | 11.5% |
|  | rs2211858 | 7896 | intron2 | C/G | 15.3% | 12.5% |
|  | rs2836163 | 14877 | intron2 | C/A | 14.8% | 11.4% |
|  | rs2154561 | 23284 | intron2 | A/G | 13.9% | 11.7% |
|  |  |  |  |  |  |  |
| EGR4 | rs1522928 | -4927 | 5'-upstream | C/T | 41.4% | 40.0% |
| (2p13) | rs2229294 | 2077 | 3’-UTR | C/T | 42.1% | 42.9% |
|  | rs7566449 | 8545 | 3'-downstream | C/T | 43.3% | 42.3% |
|  |  |  |  |  |  |  |
| FGFR3 | rs12641194 | -6849 | 5'-upstream | C/T | 30.8% | 28.5% |
| (4p16.3) | rs3135890 | 10895 | intron8 | C/T | 21.0% | 23.8% |
| FMR1NB | rs6626288 | -2723 | 5'-upstream | G/T | 38.2% | 35.0% |
| (Xq27.3-q28) | rs7881398 | 9897 | intron1 | C/T | 16.0% | 19.7% |
|  | rs5904832 | 19459 | intron1 | C/T | 16.5% | 20.3% |
|  | rs5904833 | 22298 | intron2 | A/G | 18.7% | 21.7% |
|  | rs764631 | 25399 | exon3-non-synonymous | T/C | 16.0% | 20.9% |
|  |  |  |  |  |  |  |
| GAJ | rs6535914 | -3251 | 5'-upstream | T/C | 37.5% | 34.9% |
| (4q31.3) | rs1440081 | 2277 | intron1 | C/T | 26.6% | 27.1% |
|  | rs9999685 | 19487 | intron4 | T/C | 25.6% | 24.0% |
|  | rs12499126 | 26514 | intron4 | G/A | 26.8% | 23.9% |
|  | rs12503471 | 30566 | intron4 | G/T | 26.8% | 24.2% |
|  | rs10014978 | 38747 | intron4 | G/A | 25.5% | 24.6% |
|  | rs6818341 | 45539 | intron4 | A/G | 12.6% | 12.4% |
|  | rs10023089 | 53853 | intron5 | A/T | 30.3% | 33.3% |
|  | rs12502274 | 59403 | intron6 | G/A | 28.0% | 24.4% |
|  | rs11099880 | 70372 | 3’-UTR | A/T | 28.0% | 25.1% |
|  | rs12711286 | 75930 | 3'-downstream | G/T | 28.2% | 24.7% |

Table S2. Continued

| Gene | rs ID | Sequence Position^a^ | Localization | Variation | MAF | |
| --- | --- | --- | --- | --- | --- | --- |
| (Map) |  |  |  |  | Case | Control |
| HIST1H2AA | rs4464787 | -6803 | 5'-upstream | C/G | 26.4% | 23.5% |
| (6p22.2) | rs9358871 | 80 | exon1-synonymous | A/G | 27.2% | 23.7% |
|  | rs9461204 | 8289 | 3'-downstream | A/G | 8.1% | 5.9% |
|  |  |  |  |  |  |  |
| HIST1H2BA | rs4712960 | 128 | exon1-synonymous | T/C | 27.9% | 25.6% |
| (6p22.2) | rs9358872 | 380 | exon1-synonymous | A/G | 27.3% | 23.6% |
|  |  |  |  |  |  |  |
| HNRNPG-T | rs7129581 | -3392 | 5'-upstream | T/C | 14.7% | 12.1% |
| (11p15) | rs4277091 | 7798 | 3'-downstream | T/C | 24.5% | 20.3% |
| HORMAD1 | rs12072339 | 7253 | intron5 | A/G | 40.1% | 38.1% |
| (1q21.2) | rs1336900 | 14319 | exon10-non-synonymous | G/A | 39.3% | 38.5% |
|  | rs2184833 | 17869 | intron13 | C/T | 39.0% | 38.6% |
|  | rs6700022 | 22868 | 3’-UTR | T/G | 39.3% | 37.5% |
|  | rs6587518 | 31565 | 3'-downstream | T/C | 39.4% | 37.8% |
|  |  |  |  |  |  |  |
| INHBB | rs6728617 | -9307 | 5'-upstream | A/G | 8.0% | 7.4% |
| (2q14.2) | rs7578624 | -521 | 5'-upstream | C/G | 18.8% | 19.6% |
|  | rs7581178 | 7387 | 3'-downstream | G/C | 20.3% | 20.8% |
|  |  |  |  |  |  |  |
| LOC112714 | rs7419565 | 3018 | intron2 | T/C | 38.6% | 38.4% |
| (2q21.1) |  |  |  |  |  |  |
|  |  |  |  |  |  |  |
| **LOC92196** | rs4254463 | -5901 | 5'-upstream | G/A | 19.5% | 15.0% |
| (2q24.1) | rs908404 | 2538 | intron1 | T/C | 19.3% | 14.4% |
|  | **rs9869** | 11757 | exon3-non-synonymous | C/T | **18.4%** | **13.4%** |
|  | rs10016 | 20600 | 3’-UTR | G/A | 10.8% | 8.9% |
|  |  |  |  |  |  |  |
| MGC40042 | rs5758427 | 4170 | intron2 | G/A | 6.7% | 5.3% |
| (22q13.2) | rs5758431 | 14399 | intron3 | G/A | 6.7% | 4.7% |
|  | rs5758434 | 20645 | intron6 | A/G | 6.1% | 5.3% |
|  | rs2064189 | 32481 | intron9 | C/T | 6.1% | 5.2% |
|  | rs7291082 | 51661 | intron15 | G/A | 35.2% | 35.8% |
|  | rs6519287 | 73751 | intron20 | T/G | 35.9% | 36.9% |
|  | rs8136343 | 87950 | intron26 | C/T | 28.2% | 30.3% |
|  | rs4822052 | 98223 | intron30 | G/A | 6.8% | 5.6% |
| MTL5 | rs4930608 | -12594 | 5'-upstream | C/T | 10.7% | 9.9% |
| (11q13.2-q13.3) | rs3019598 | -8598 | 5'-upstream | G/A | 18.9% | 15.4% |
|  | rs3019598 | -8598 | 5'-upstream | G/A | 18.9% | 15.4% |
|  | rs2278908 | -4908 | 5'-upstream | G/T | 20.9% | 19.6% |
|  | rs7103254 | 13938 | intron6 | G/A | 23.7% | 19.8% |
|  | rs7942697 | 23540 | intron6 | G/T | 17.1% | 15.1% |
|  | rs7101673 | 29702 | intron6 | T/C | 23.7% | 20.2% |
|  |  |  |  |  |  |  |
| **NYD-SP20** | **rs3829957** | -3785 | 5'-upstream | C/T | **42.1%** | **36.2%** |
| (17p13.3) | **rs2318035** | 12022 | intron5 | A/G | **42.9%** | **36.1%** |
|  | **rs1488689** | 22797 | exon6-non-synonymous | A/G | **41.6%** | **35.3%** |
|  | **rs17822627** | 31572 | exon9-synonymous | T/C | **41.3%** | **34.7%** |
|  | **rs2318033** | 40646 | 3'-downstream | A/T | **42.9%** | **36.8%** |
|  |  |  |  |  |  |  |
| OTEX | rs6646573 | -43 | 5'-upstream | G/A | 36.2% | 40.0% |
| (Xq24) | rs6646569 | 8073 | 3'-downstream | C/T | 36.2% | 40.5% |

Table S2. Continued

| Gene | rs ID | Sequence Position^a^ | Localization | Variation | MAF | |
| --- | --- | --- | --- | --- | --- | --- |
| (Map) |  |  |  |  | Case | Control |
| PAGE1 | rs6610023 | -7142 | 5'-upstream | G/A | 8.0% | 11.0% |
| (Xp11.23) | rs6610017 | 3532 | intron3 | C/T | 16.2% | 13.5% |
|  | rs6608911 | 10308 | 3'-downstream | C/T | 7.0% | 11.9% |
|  |  |  |  |  |  |  |
| **PAGE5** | **rs2148982** | 2260 | intron3 | C/T | **30.1%** | **24.5%** |
| (Xp11.21) | **rs5913800** | 5332 | 3'-downstream | A/G | **29.5%** | **22.3%** |
|  | **rs5914276** | 8924 | 3'-downstream | C/G | **29.9%** | **23.7%** |
|  |  |  |  |  |  |  |
| PIK3R3 | rs2486445 | -6902 | 5'-upstream | T/C | 32.9% | 36.1% |
| (1p34.1) | rs1707302 | -2450 | 5'-upstream | C/T | 29.5% | 31.9% |
|  | rs785495 | 10580 | intron1 | C/T | 36.4% | 37.1% |
|  | rs785490 | 21343 | intron1 | C/T | 30.7% | 31.0% |
|  | rs785520 | 29631 | intron1 | C/T | 36.1% | 38.4% |
|  | rs809774 | 38223 | intron1 | A/T | 37.6% | 35.9% |
|  | rs4660910 | 50065 | intron1 | T/G | 33.7% | 38.1% |
|  | rs809775 | 60427 | intron3 | A/T | 34.7% | 34.3% |
|  | rs2458400 | 67669 | intron5 | T/C | 30.5% | 34.0% |
|  | rs785467 | 76908 | exon7-non-synonymous | A/T | 30.0% | 32.9% |
|  | rs1768817 | 83933 | intron7 | C/G | 29.8% | 33.2% |
|  | rs1707337 | 89698 | 3’-UTR | C/T | 30.0% | 32.7% |
|  | rs1768809 | 95631 | 3'-downstream | G/T | 31.5% | 36.0% |
|  |  |  |  |  |  |  |
| RAD54L | rs12141269 | -8312 | 5'-upstream | C/T | 15.4% | 15.6% |
| (1p32) | rs9793263 | 8971 | intron3 | G/A | 32.9% | 34.6% |
|  | rs4660917 | 17435 | intron8 | A/T | 22.1% | 22.3% |
|  | rs1048771 | 30482 | 3’-UTR | C/T | 34.3% | 32.2% |
|  |  |  |  |  |  |  |
| SPAG5 | rs2302206 | 13601 | intron6 | G/T | 18.9% | 17.3% |
| (17q11.2) | rs652677 | 21780 | 3’-UTR | G/T | 18.8% | 21.4% |
|  |  |  |  |  |  |  |
| SSX6 | rs545573 | -7484 | 5'-upstream | G/A | 42.9% | 44.6% |
| (Xp11.2) | rs2872560 | 15362 | 3'-downstream | T/C | 45.4% | 46.4% |
|  |  |  |  |  |  |  |
| STAG3 | rs3735242 | -3499 | 5'-upstream | C/T | 32.6% | 36.3% |
| (7q22.1) | rs6948729 | 12024 | intron8 | G/A | 41.3% | 43.0% |
|  | rs1043915 | 24497 | exon24-synonymous | T/A | 41.6% | 42.7% |
|  | rs1061230 | 31917 | intron29 | G/A | 43.1% | 45.6% |
|  | rs1727133 | 37522 | 3'-downstream | C/T | 41.6% | 42.7% |
| SYCP3 | rs7135472 | -4514 | 5'-upstream | C/T | 37.7% | 34.4% |
| (12q23.2) | rs3751248 | 1683 | intron1 | C/A | 36.2% | 36.3% |
|  | rs10860779 | 5985 | intron6 | C/A | 36.5% | 33.8% |
|  | rs10083157 | 14233 | 3'-downstream | T/G | 30.4% | 29.8% |
|  |  |  |  |  |  |  |
| **TEX14** | rs686425 | -7314 | 5'-upstream | G/A | 47.0% | 46.0% |
| (17q22) | rs302874 | 1420 | intron1 | C/T | 47.2% | 46.2% |
|  | rs302865 | 12439 | intron1 | C/T | 45.0% | 44.4% |
|  | rs446613 | 19870 | intron1 | A/C | 47.4% | 50.3% |
|  | rs1631237 | 34721 | intron1 | C/T | 46.3% | 47.3% |
|  | **rs302843** | 41430 | intron2 | A/G | **44.8%** | **50.5%** |
|  | rs2611782 | 51460 | intron2 | C/T | 46.3% | 47.8% |
|  | rs591200 | 63515 | intron2 | C/T | 40.2% | 43.0% |

Table S2. Continued

| Gene | rs ID | Sequence Position^a^ | Localization | Variation | MAF | |
| --- | --- | --- | --- | --- | --- | --- |
| (Map) |  |  |  |  | Case | Control |
| (TEX14) | rs591200 | 63515 | intron2 | C/T | 40.2% | 43.0% |
|  | rs9898626 | 71197 | intron5 | C/G | 47.1% | 44.4% |
|  | rs302854 | 85804 | intron10 | T/C | 46.3% | 47.3% |
|  | rs8072873 | 100367 | intron15 | G/C | 24.7% | 27.9% |
|  | rs6503870 | 110398 | exon20-non-synonymous | T/C | 46.6% | 47.0% |
|  | **rs1267542** | 114726 | intron22 | T/C | **45.7%** | **52.4%** |
|  | rs3803751 | 119060 | intron24 | T/C | 24.6% | 28.4% |
|  | rs1267545 | 122507 | intron26 | A/G | 45.6% | 49.2% |
|  | **rs1974586** | 128685 | intron29 | C/T | **24.6%** | **30.7%** |
|  | rs2333332 | 138362 | 3'-downstream | C/T | 48.6% | 49.7% |
|  | rs714959 | 140815 | 3'-downstream | T/C | 21.6% | 18.3% |
|  | **rs12453459** | 145125 | 3'-downstream | T/C | **47.7%** | **41.2%** |
|  |  |  |  |  |  |  |
| **TKTL1** | rs631 | -8147 | 5'-upstream | G/A | 23.1% | 19.0% |
| (Xq28) | **rs6655282** | 12986 | intron6 | G/A | **11.2%** | **4.9%** |
|  | rs766420 | 20834 | intron9 | C/G | 22.5% | 20.0% |
|  | rs2872817 | 24848 | 3’-UTR | A/G | 26.7% | 23.2% |
|  |  |  |  |  |  |  |
| VCX | rs5936101 | 4679 | 3'-downstream | G/A | 47.3% | 49.7% |
| (Xp22) |  |  |  |  |  |  |
|  |  |  |  |  |  |  |
| VCX2 | rs12395480 | -2816 | 5'-upstream | C/T | 16.4% | 20.0% |
| (Xp22.32) |  |  |  |  |  |  |
|  |  |  |  |  |  |  |
| XAGE3 | rs5943751 | 5535 | 3’-UTR | T/C | 35.5% | 39.5% |
| (Xp11.22-p11.21) |  |  |  |  |  |  |
|  |  |  |  |  |  |  |
| **XAGE5** | **rs4543711** | 5279 | intron4 | A/G | **5.3%** | **11.0%** |
| (Xp11.22) |  |  |  |  |  |  |

For SNPs with discrepancies in MAFs of 5% or greater between cases and controls, gene symbols haboring the SNPs and their SNP IDs are shown in *boldface*.

^a^ Nucleotide position from the first nucleotide of exon 1 of each gene
